# Proteomic insights into modifiable risk of venous thromboembolism and cardiovascular comorbidities

**DOI:** 10.1016/j.jtha.2023.11.013

**Published:** 2023-11-27

**Authors:** Shuai Yuan, Fengzhe Xu, Han Zhang, Jie Chen, Xixian Ruan, Yuying Li, Stephen Burgess, Agneta Åkesson, Xue Li, Dipender Gill, Susanna C. Larsson

**Affiliations:** 1 Unit of Cardiovascular and Nutritional Epidemiology, Institute of Environmental Medicine, Karolinska Institute, Stockholm, Sweden; 2 Key Laboratory of Growth Regulation and Translational Research of Zhejiang Province, School of Life Sciences, Westlake University, Hangzhou, China; 3 Department of Big Data in Health Science School of Public Health, Center of Clinical Big Data and Analytics of the Second Affiliated Hospital, Zhejiang University School of Medicine, Hangzhou, China; 4 Department of Gastroenterology, the Third Xiangya Hospital, Central South University, Changsha, China; 5 Department of Medical Epidemiology and Biostatistics, Karolinska Institute, Stockholm, Sweden; 6 Medical Research Council Biostatistics Unit, University of Cambridge, Cambridge, UK; 7 Department of Public Health and Primary Care, University of Cambridge, Cambridge, UK; 8 Department of Epidemiology and Biostatistics, School of Public Health, Imperial College London, London, UK; 9 Unit of Medical Epidemiology, Department of Surgical Sciences, Uppsala University, Uppsala, Sweden

**Keywords:** modifiable, proteomics, venous thrombosis

## Abstract

**Background:**

Venous thromboembolism (VTE) has been associated with several modifiable factors (MFs) and cardiovascular comorbidities. However, the mechanisms are largely unknown.

**Objectives:**

We aimed to decipher proteomic pathways underlying the associations of VTE with MFs and cardiovascular comorbidities.

**Methods:**

A 2-stage network Mendelian randomization analysis was conducted to explore the associations between 15 MFs, 1151 blood proteins, and VTE using data from a genome-wide meta-analysis including 81 190 cases of VTE. We used protein data from 35 559 individuals as the discovery analysis, and from 2 independent studies including 10 708 and 54 219 participants as the replication analyses. Based on the identified proteins, we assessed the druggability and examined the cardiovascular pleiotropy.

**Results:**

The network Mendelian randomization analyses identified 10 MF–VTE, 86 MF–protein, and 34 protein–VTE associations. These associations were overall consistent in the replication analyses. Thirty-eight pathways with directionally consistent direct and indirect effects in the MF–protein–VTE pathway were identified. Low-density lipoprotein receptor–related protein 12 (LRP12: 34.3%-58.1%) and coagulation factor (F)XI (20.6%-39.6%) mediated most of the associations between 3 obesity indicators and VTE. Likewise, coagulation FXI mediated most of the smoking–VTE association (40%; 95% CI, 20%-60%) and insomnia–VTE association (27%; 95% CI, 5%-49%). Many VTE-associated proteins were highly druggable for thrombotic conditions. Five proteins (interleukin-6 receptor subunit alpha, LRP12, prothrombin, angiopoietin-1, and low-density lipoprotein receptor–related protein 4) were associated with VTE and its cardiovascular comorbidities.

**Conclusion:**

This study suggests that coagulation FXI, a druggable target, is an important mediator of the associations of obesity, smoking, and insomnia with VTE risk.

## 1 Introduction

Venous thromboembolism (VTE) affects 1 to 2 individuals per 1000 in Europe and the United States and has been ranked at the top among vascular diseases with regard to incidence, morbidity, and mortality [1]. Although multifactorial in etiology, the risk of VTE has been fortunately found to be modifiable [1]. Mainly for unprovoked VTE, reducing obesity [2] and adopting a healthy lifestyle [3] appear to be associated with a lower risk of VTE. Even though the classic Virchow triad theory may help clarify underlying mechanisms behind these associations, data are scarce to illuminate the molecular pathways linking these modifiable factors to VTE. Given that blood proteins have been revealed in thrombosis and coagulation [4,5] and may be altered by obesity and lifestyle factors [6], we hypothesize that the modifiable risk of VTE may be partly attributed to changes in proteomic features. A clear and comprehensive appraisal of these pathways not only benefits the understanding of the pathogenesis of VTE but also provides evidence support for better disease prevention and management in a precision manner.

In clinical practice, antithrombotic therapy is used for treating VTE. Despite being effective, the risk of major bleeding during antithrombotic treatment has been found to be considerable [7]. In addition, no current tools can precisely predict bleeding risk [8]. Thus, identifying new targets for attenuating thrombosis with the potential for less bleeding is of great importance for VTE drug development. Blood proteins, usually the principal regulators of molecular pathways, are always treated as therapeutic targets [9]. By conducting a comprehensive investigation on protein–VTE associations, this study aimed to pinpoint potential drug targets by integrating clinical, genetic, and omics data from large databases of human data. Cardiovascular risk has been found to be increased among patients with VTE [10]. Thus, we also aimed to clarify the proteomic etiology of this.

Mendelian randomization (MR) analysis is an epidemiological approach that can reinforce association inference using observational genetic data [11]. Leveraging genetic variants as an instrumental variable (IV) for exposure, the MR design can minimize confounding since genetic variants are randomly assorted at conception and assumed to be unassociated with confounders [11]. This process mimics the randomization process in randomized controlled trials. The MR design can also diminish reverse causation as germline genotypes cannot be modified by the onset or progression of disease [11]. There are 3 important assumptions of MR. First, the genetic variants used as the IVs should be strongly associated with the exposure (eg, blood proteins and modifiable factors in this study). Second, the genetic instruments should not be associated with any confounders. Third, the genetic variants used as the IVs should influence the outcome only through the exposure not via other alternative pathways. Violation of the third assumption is known as pleiotropy, among which the unbalanced horizontal type is a major challenge for MR studies [12]. Regarding investigations on proteome–disease associations, MR design has been usually observed to satisfy key assumptions, particularly when using *cis*-variants in protein-encoding genes as genetic instruments for the proteins [9,13]. For phenotypic traits with multiple genetic variants as the IVs, several methods have been developed to generate indication of horizontal pleiotropy and to minimize this by removing pleiotropic genetic instruments [14]. Overall, considering the merits of MR design as well as genetic data available for modifiable factors and blood proteins, here, we conducted this study to disentangle the proteomic pathways linking modifiable factors to VTE as well as to explore shared protein etiologies between VTE and its cardiovascular comorbidities.

## 2 Methods

### 2.1 Study design

**Figure 1:**
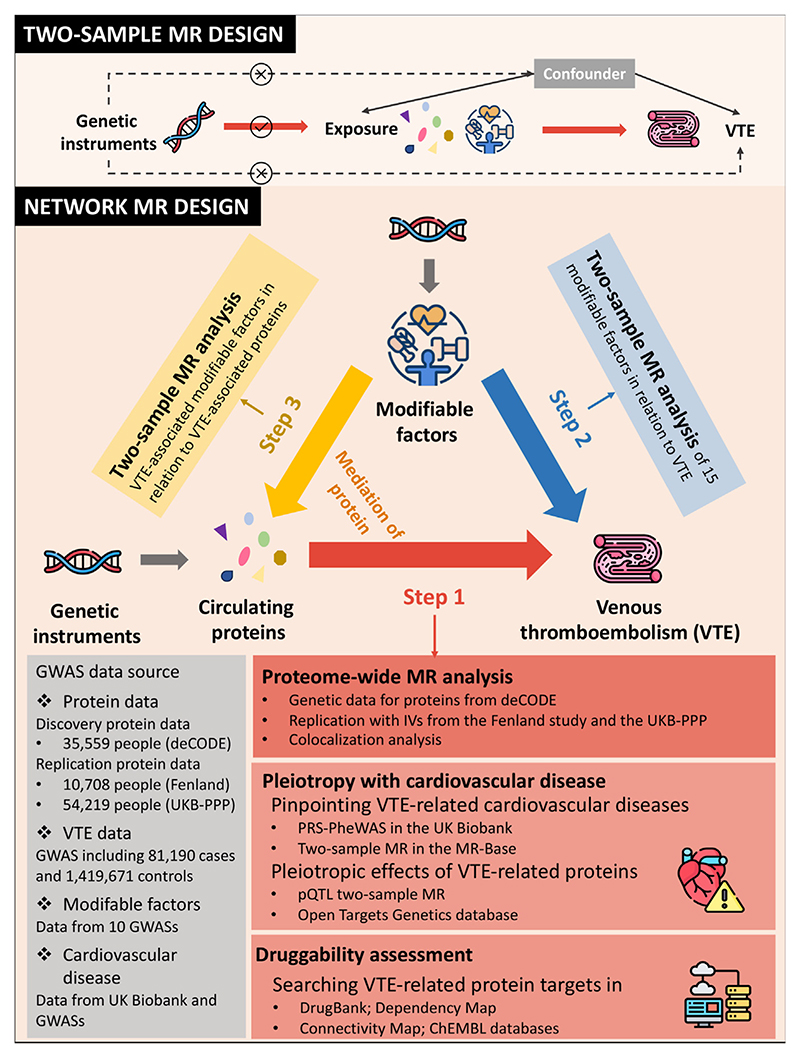
The schematic study design of traditional and network Mendelian randomization (MR) approaches. GWAS, genome-wide association study; PQTL, proteomic quantitative trait locus; PRS-PheWAS, polygenic risk score–phenome-wide association study; UKB-PPP, UK Biobank Pharma Proteomics Project; VTE, venous thromboembolism.

Figure 1 shows the schematic study design of traditional and network MR approaches. In this study, we first conducted proteome-wide MR and colocalization analyses to identify VTE-associated blood proteins.

In the proteome-wide MR on VTE, we first selected suitable genetic variants as IVs to mimic the levels of blood proteins with data from the corresponding protein genome-wide association studies (GWASs) and then used the MR approach to estimate the associations between a wide range of blood proteins and the risk of VTE. Likewise, we performed MR analyses to explore the VTE-associated modifiable factors and further the associations between VTE-associated modifiable factors and VTE-associated blood proteins. Based on this 2-stage network MR design, we estimated mediation effects of proteins in the associations between modifiable factors and VTE. We performed reverse MR analysis to rule out reverse causation and conducted replication analyses using data from independent protein GWASs. Based on identified proteins, we performed 2 downstream analyses: 1) druggability assessment and 2) pleiotropic associations with cardiovascular comorbidities. The study used publicly available data generated in published studies (Supplementary Table S1). These studies had been approved by the corresponding ethical committee, and the participants signed informed consent forms.

### 2.2 Blood protein data

In the discovery analysis, also called primary analysis, summary-level data for 4907 blood proteins were obtained from GWASs in 35 559 Icelanders (mean age, 55 years; 50% women) [15]. Plasma proteins were measured using the SomaScan version 4 assay (SomaLogic). Information on genotyping, imputation, and quality control is described in detail in the original paper [15]. The levels of proteins were under the rank-inverse normal transformation by age, sex, and sample age and standardized. Associations of genetic variants with protein levels were estimated using the linear mixed model. For MR analysis, we selected index cis–single nucleotide polymorphisms (SNPs) associated with protein levels at the genome-wide significance level (*P* < 5 × 10^−8^) as the IVs for proteins. Regarding identified proteins associated with VTE in the discovery analysis, we used 2 independent protein GWAS sources, the Fenland study and the UK Biobank Pharma Proteomics Project (UKB-PPP), for replication. GWAS summary-level data and IVs were extracted from the Fenland study including 10 708 participants, whose blood proteins were profiled using the SomaScan version 4 assay [16]. We additionally selected IVs from a GWAS in the UKB-PPP (*N* = 54 219) [17] to confirm the association where available. In UKB-PPP, proteomic profiling was performed by the Olink platform (Olink Explore 3072 platform). In both replication data, the IVs were selected under the same criteria as previously described.

### 2.3 VTE data

Summary-level data on VTE were extracted from a genome-wide meta-analysis of 81 190 cases and 1 419 671 controls of European ancestry from 6 cohorts [18]. VTE cases were defined by hospital or register records (International Classification of Diseases, 9th or 10th Revision). Detailed information on genotyping, imputation, and quality control at the participant level and gene level is described somewhere else [18]. Associations of DNA variants with odds of VTE were estimated by logistic regression with at least age (or year of birth), sex, and principal components as covariates. We selected SNPs strongly associated with VTE at the genome-wide significance level (*P* < 5 ×10^−8^) and then clumped these selected SNPs by setting the linkage disequilibrium *r*^2^ threshold at 0.01, which lead to the remaining SNPs as the IVs to proxy the genetic liability to VTE in the reverse MR analysis.

### 2.4 Modifiable factor data

Our study primarily focused on understanding the links between previously identified and potentially modifiable risk factors, notably obesity and lifestyle habits, and VTE susceptibility, which thus included adiposity indicators (body mass index, waist-to-hip ratio, and visceral adiposity) [2] and lifestyle factors (smoking initiation [19], lifetime smoking index [19], alcohol intake [20], coffee and caffeine consumption [21], moderate-to-vigorous physical activity [22], and leisure screen time [23]). Recognizing the American Heart Association’s emphasis on sleep as a pivotal factor for cardiometabolic health [24], we also incorporated sleep-related traits [25], including sleep duration, short and long sleep duration, daytime napping, and insomnia. Data sources for included modifiable traits are clarified in Supplementary Table S1. To select genetics IVs, we first extracted SNPs associated with each trait at *P* < 5 × 10^−8^ from the corresponding GWAS. SNPs with high linkage disequilibrium (*r*^2^ > 0.01) were pruned and the SNP in linkage disequilibrium with the lowest *P* value was retained. The remaining SNPs were selected as the IVs to proxy the effects of the above modifiable factors.

### 2.5 Druggability assessment of VTE-associated proteins

To assess the druggability of the identified VTE-associated proteins, we searched the DrugBank, Dependency Map, Connectivity Map, and ChEMBL databases. We documented information on drug name and the process of drug development, and classified these protein targets into 4 classes: 1) approved (at least 1 drug targeting the protein has been approved), 2) in clinical trials (at least 1 drug targeting the protein is currently studied in clinical trials), 3) preclinical (at least 1 drug targeting the protein is in preclinical pipelines), 4) druggable (proteins cannot be identified in drug databases but listed as druggable targets), and 5) currently not listed as druggable.

### 2.6 Pleiotropic effects of VTE-associated proteins on cardiovascular comorbidities

To explore VTE-associated cardiovascular comorbidities, we performed a polygenic risk score–phenome-wide association study (PRS-PheWAS) in the UK Biobank study. The IVs (*P* < 5 × 10^−8^ and *r*^2^ < 0.01) for VTE were selected from the genome-wide meta-analysis of 81 190 cases and 1 419 671 controls [18]. We included a wide range of clinical outcomes in this analysis and aimed to explore which systems are most influenced by genetic susceptibility to VTE, which may provide support for selecting major comorbidities in the following analysis. Detailed descriptions for polygenic risk score construction and PRS-PheWAS analysis are described in Supplementary Methods. We also used the MR-Base (a database and analytical platform for MR, https://www.mrbase.org/) to confirm the associations of VTE with common cardiovascular diseases, including coronary artery disease, myocardial infarction, ischemic stroke, atrial fibrillation, heart failure, and peripheral artery disease. Data sources for these outcomes in the MR-Base can be found in Supplementary Table S1. We performed MR analysis to examine the associations of VTE-associated proteins with these cardiovascular comorbidities, summarized cardiovascular effects of proteins shared by VTE, and studied cardiovascular comorbidities using the Open Targets Genetics database [26].

### 2.7 Statistical analysis

The *F* statistic was calculated for each protein to measure the strength of used IV. Proteins without SNPs in the VTE dataset or with IV of *F* < 10 were removed from the analysis. For the MR analysis of protein–VTE associations, the odds ratio and corresponding CI of the association were estimated by the Wald ratio test and the delta method, respectively. For the MR analysis of modifiable risk factors and reverse MR analysis, we used the inverse variance weighted method under the multiplicative random effects as the primary analysis, which was supplemented by 3 sensitivity analyses, including the weighted median, MR-Egger, and MR-PRESSO, to examine the robustness of the results and detect potential horizontal pleiotropy. For mediation estimation, the proportion mediated by a protein was calculated as the estimated effect of the modifiable factor on protein levels multiplied by the estimated effect of protein levels on VTE. The propagation of error method (also known as the delta method) was employed to estimate the SE associated with the mediation effect [27]. The method is based on the principle that errors in measurements or estimates can propagate and influence the precision of derived quantities. In the context of this MR analysis, the propagation of error method provides uncertainty estimates surrounding the mediation effect of protein in the association between modifiable risk factors and VTE risk. For proteins, we performed colocalization analysis based on a Bayesian model to test whether the protein and VTE share the same causal variant in the encoding gene region (Supplementary Methods) and used a web tool (https://genemania.org/) to map the network among identified proteins [28]. In the primary analysis, we used the Bonferroni method or false discovery rate (Benjamini–Hochberg method) correction to adjust for multiple testing. In the replication analysis, the association with the *P* value of <.05 was deemed significant. The analyses were performed using TwoSampleMR, MendelianRandomization, and coloc packages in R software (4.4.1; R Core Team).

## 3 Results

### 3.1 Proteome-wide MR analysis identified 34 plasma proteins associated with VTE

**Figure 2:**
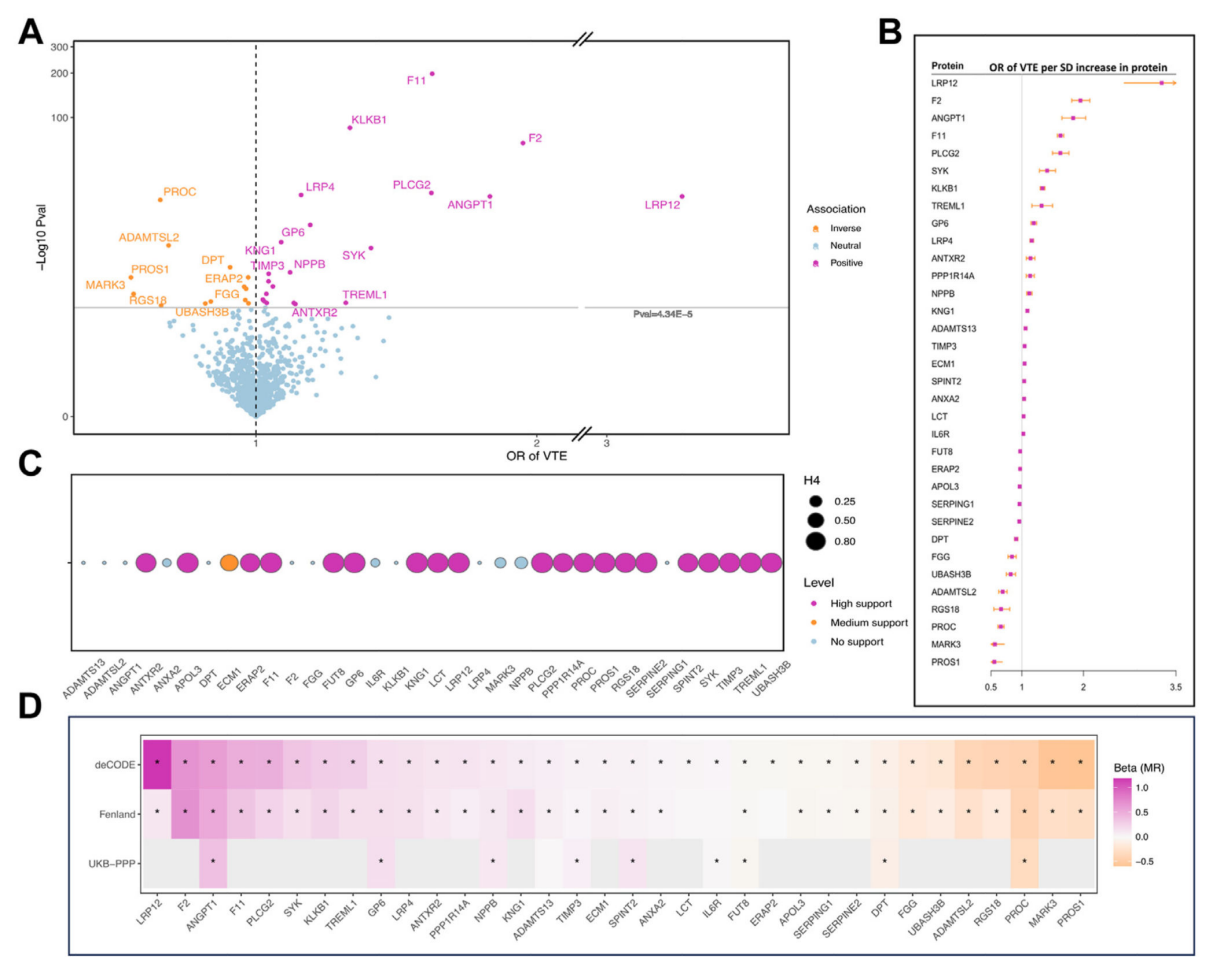
Mendelian randomization (MR) and colocalization analyses on the associations between blood proteins and the risk of venous thromboembolism (VTE). (A) The volcano plot of results of the proteome-wide MR analysis of VTE using the discovery deCODE protein data. (B) Forest plot of identified MR associations between blood proteins and VTE risk using the discovery deCODE protein data. (C) Results of colocalization analysis based on deCODE protein data. (D) Comparison of associations in the discovery analysis based on deCODE protein data and replication analyses based on Fenland and UKB-PPP protein data. The protein–VTE association successfully replicated using genetic instruments from Fenland or UK Biobank Pharma Proteomics Project (UKB-PPP) is marked by an asterisk. Full names of the proteins listed can be found in Supplementary Table S2. OR, odds ratio.

After removing proteins without SNPs in the outcome data or with weak instruments (*F* statistic < 10), the proteome-wide MR analysis included a total of 1151 plasma proteins. The results of all 1151 proteins are shown in Supplementary Table S2. After Bonferroni correction, genetically predicted levels of 34 proteins were identified to be associated with VTE (*P* < .05/1151; Figure 2A). Per SD increase in genetically predicted protein levels, the odds ratio of VTE ranged from 0.55 (95% CI: 0.45-0.68) for PROS1 (protein S) to 3.27 (95% CI: 2.66-4.01) for low-density lipoprotein receptor–related protein 12 (LPR12) (Figure 2B). Among 34 protein–VTE associations, 23 had strong colocalization support with a PH4 of >0.8 and 1 association had medium colocalization support with 0.8 > PH4 > 0.5 (Figure 2C). Colocalization analysis additionally identified 9 proteins with strong colocalization support with VTE albeit without MR support (Supplementary Table S3). The network of VTE-associated proteins is shown in Supplementary Figure S1. Among these 34 proteins, 34 and 10 proteins had IVs from the Fenland study and UKB-PPP, respectively. We could not obtain IVs for the rest 24 proteins in UKB-PPP due to a smaller number of proteins measured by Olink in this study. For proteins with available IVs, we replicated 31 (91.2%) associations using IVs for protein from the Fenland study and 9 (90%) associations using IVs for proteins from the UKB-PPP (Figure 2D, Supplementary Tables S4 and S5). In the reverse MR analysis, we found no evidence of associations of genetic liability to VTE with the levels of identified blood proteins after multiple testing correction (Supplementary Table S6).

### 3.2 More than 10 proteins had a high potential of druggability

We collected data on clinical trials in 4 drug databases for drugs targeting VTE-associated proteins identified in the proteome-wide MR analysis to reveal the druggability. We found 12 proteins being targets for drugs approved, and 9 of them were used to treat thrombotic conditions (Supplementary Table S7). Most of these proteins were regarded druggable but in different stages of druggability exploration.

### 3.3 Ten modifiable factors were associated with VTE

**Figure 3:**
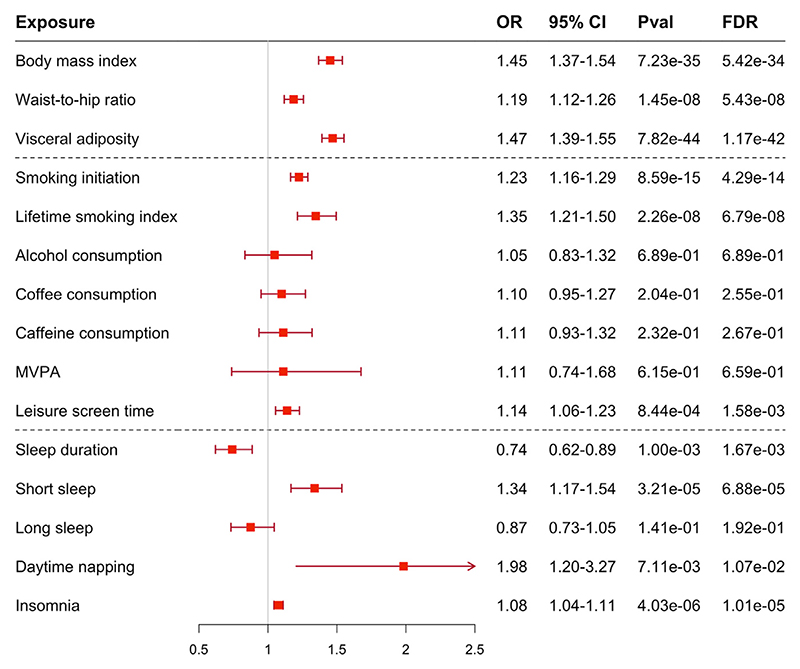
Associations of modifiable factors with the risk of venous thromboembolism. FDR, false discovery rate; MVPA, moderate-to-vigorous physical activity; OR, odds ratio.

Genetically proxied 10 of 15 modifiable factors were associated with VTE after Bonferroni correction (Figure 3). Genetic predisposition to obesity, cigarette smoking, sedentary lifestyle, short sleep duration, daytime napping, and insomnia was associated with an increased risk of VTE (Figure 3). The associations were consistent in sensitivity analyses (Supplementary Table S8). Heterogeneity was observed in most associations; however, limited indication of horizontal pleiotropy was observed by MR-Egger intercept test (*P* > .05; Supplementary Table S8). We observed no evidence of associations of genetic liability to VTE with identified modifiable risk factors in the reverse MR analysis (Supplementary Table S9).

### 3.4 Associations between VTE-associated modifiable factors and VTE-associated proteins

In the MR analyses on the associations between VTE-associated modifiable factors and VTE-associated proteins, we set the significance level at the nominal level to reveal as many potential mediation signals as possible. In total, 86 pairs of associations were identified (Supplementary Table S10). Among VTE-associated modifiable factors, genetically predicted obesity indicators were associated with 28 VTE-associated proteins. Among VTE-associated proteins, there were genetically predicted 7 modifiable factors associated with ADAMTS-like protein 2, 6 with anthrax toxin receptor 2, and 5 with coagulation factor (F)XI. Most of these associations were observed to be at least directionally consistent in the replication analysis using protein data from the Fenland study (Supplementary Table S11).

### 3.5 Mediation of proteins in the associations between modifiable factors and VTE

**Figure 4:**
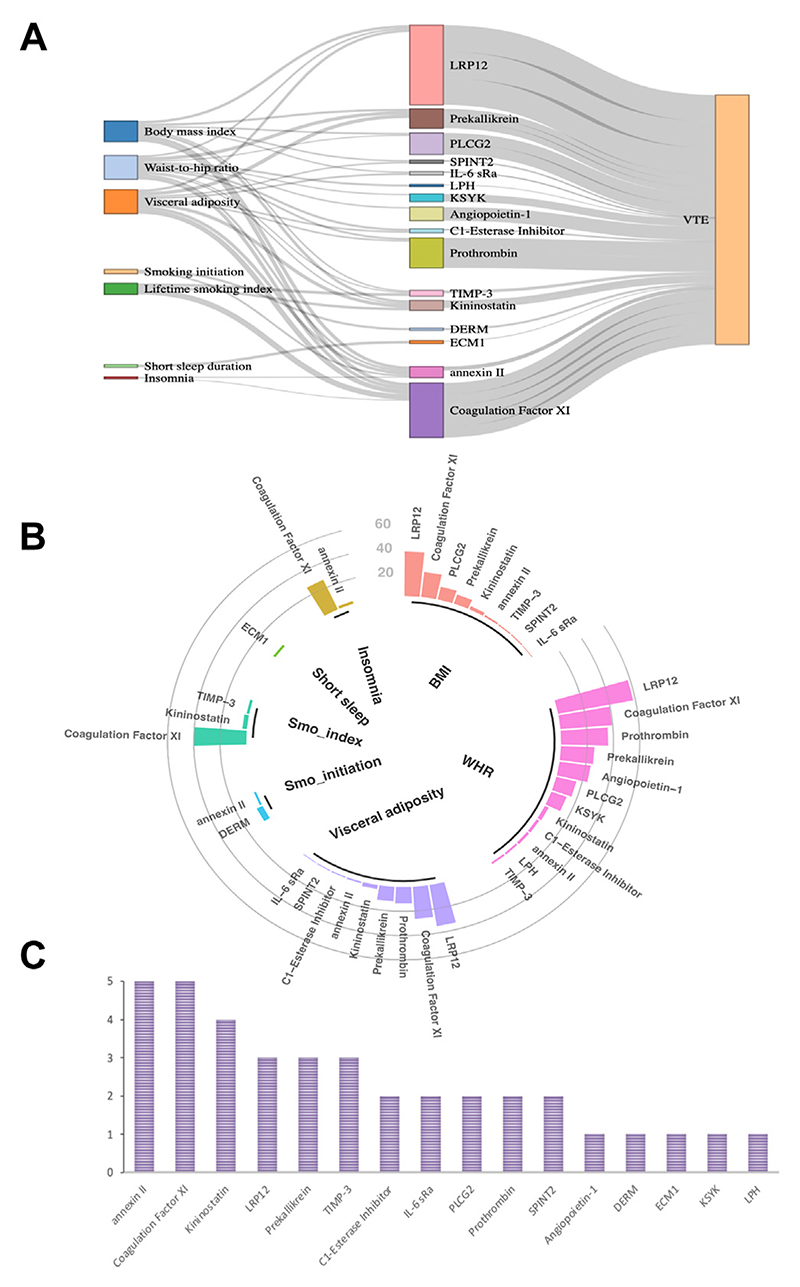
Mediation effects of proteins in the associations between modifiable factors and venous thromboembolism (VTE) risk. (A) Protein pathways linking obesity, smoking, and sleep-related traits to VTE. (B) The proportion of association between the modifiable factor and VTE mediated by a protein. (C) The count of protein mediators among all identified pathways. Full names of the proteins listed can be found in Supplementary Table S2. BMI, body mass index; Smo, smoking; WHR, waist-to-hip ratio.

We estimated mediation of 38 modifiable factor–protein–VTE combinations where the direction of the total effect (beta of the modifiable factor–VTE association) was in line with the direction of the effect through the mediator (beta of the modifiable factor–protein association × beta of the protein–VTE association) (Supplementary Table S12). Thirty of 38 combinations were related to obesity indicators, and 5 proteins (annexin II [Annexin A2], coagulation FXI, Kininostatin-1, LRP12, and prekallikrein [plasma kallikrein]) showed consistent mediation effects on the associations of 3 obesity indicators with VTE risk (Figure 4A). We observed networks (eg, coexpression, physical interactions, and pathway) between these proteins mediating the obesity–VTE association (Supplementary Figure S1). Concerning the magnitude of mediation, LPR12, coagulation FXI, and prothrombin ranked at the top for the association between obesity and VTE (Figure 4B). Genetically predicted levels of 5 proteins mediated the association between cigarette smoking and VTE, with the highest mediation for coagulation FXI (40%; 95% CI, 20%-60%; Figure 4B). Likewise, genetically predicted levels of 3 proteins mediated the association between sleep-related traits and VTE with the highest mediation for coagulation FXI (27%; 95% CI, 5%-49%; Figure 4B). Overall, genetically predicted levels of annexin II and coagulation FXI mediated the most associations between studied modifiable factors and VTE (Figure 4C). There were limited networks between annexin II and coagulation FXI (Supplementary Figure S1).

### 3.6 Pleiotropic effects of VTE-associated proteins on cardiovascular comorbidities

**Figure 5:**
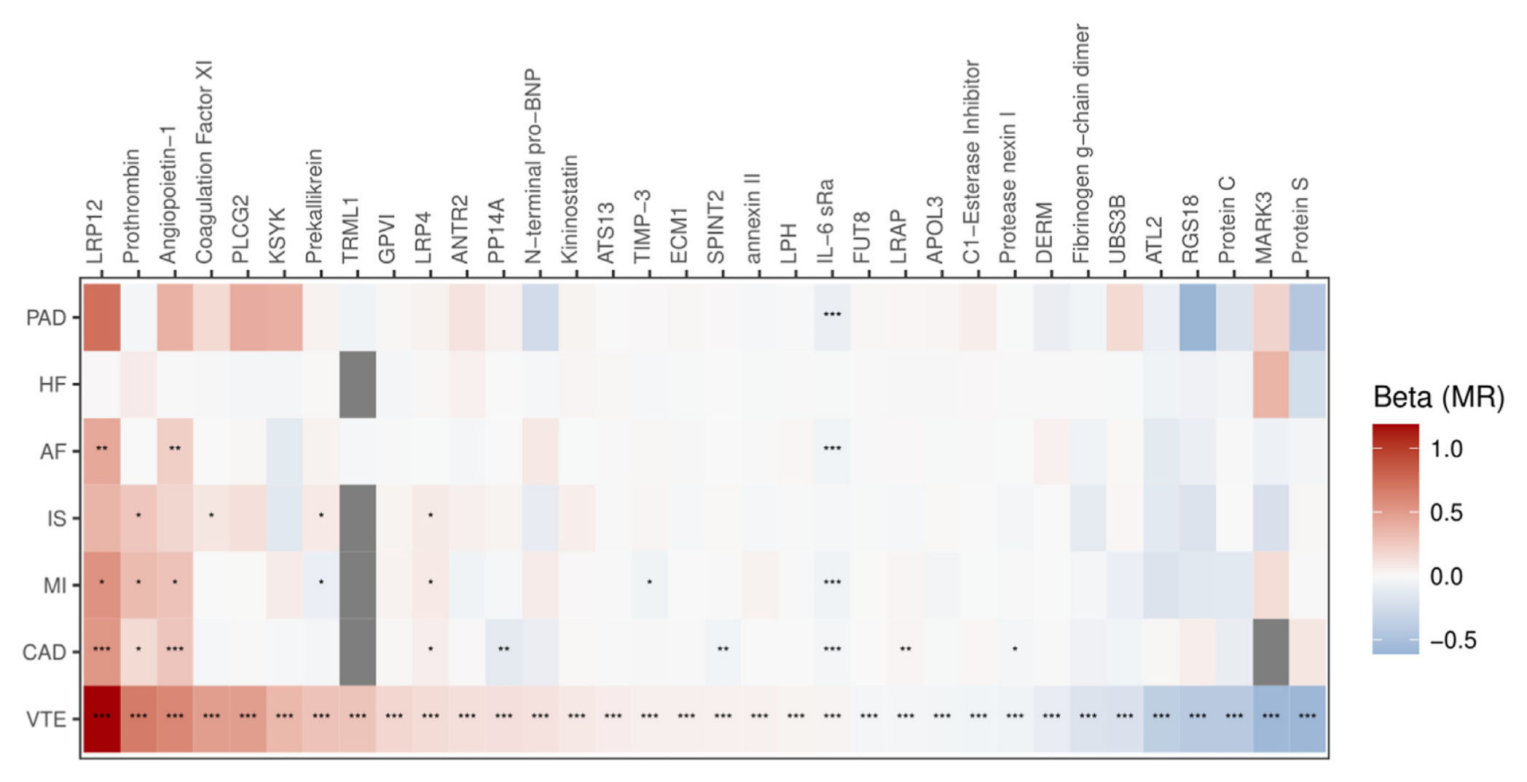
Cardiovascular pleiotropy of 34 venous thromboembolism (VTE)–associated proteins. Gray squares indicate missing values. False discovery rate (FDR) correction was used in this analysis for each outcome. ***FDR < 0.001; **0.001 < FDR < 0.01; *0.01 < FDR < 0.05. Full names of the proteins listed can be found in Supplementary Table S2. AF, atrial fibrillation; CAD, coronary artery disease; HF, heart failure; IS, ischemic stroke; MI, myocardial infarction; MR, Mendelian randomization; PAD, peripheral artery disease.

We identified cardiovascular comorbidities associated with VTE by PRS-PheWAS and MR analyses (Supplementary Figure S2 and Supplementary Table S13). In the further analysis on the association between VTE-associated proteins and cardiovascular comorbidities, genetically predicted levels of interleukin-6 receptor subunit alpha (IL-6 sRa) were inversely associated with VTE and 4 cardiovascular diseases (Figure 5). Genetically predicted levels of LRP12, prothrombin, angiopoietin-1 (ANGPT1), and low-density lipoprotein receptor–related protein 4 (LRP4) were positively associated with VTE and 3 cardiovascular diseases (Figure 5). The genetically predicted above 5 proteins were also associated with other cardiovascular diseases, with the direction being mostly consistent with the associations for VTE (Supplementary Figure S3).

## 4 Discussion

In this study, we first performed a protein-wide MR analysis on VTE, which identified genetically predicted levels of >30 blood proteins with potential roles in the development of VTE. We then conducted a 2-stage network MR analysis to explore the protein pathways linking modifiable factors to VTE. We found that genetically predicted levels of several proteins, in particular annexin II and coagulation FXI, mediated the MR associations of obesity, smoking, and insomnia with VTE. Many VTE-associated proteins were found to be highly druggable, with effects on coagulation-related conditions. We also revealed that LRP12, prothrombin, ANGPT1, LRP4, and IL-6 sRA were commonly associated with VTE and its cardiovascular comorbidities, which implies the shared etiologies between them. Our findings were overall consistent across the analyses using data from independent protein GWASs, and limited reverse causality was observed. Taken together, our findings may facilitate the understanding of the protein pathogenesis of VTE and better guide VTE prophylaxis among feathered populations, such as obese individuals and smokers. The shared proteins between VTE and its cardiovascular comorbidities may identify therapeutic targets across the spectrum of VTE-associated cardiovascular multimorbidity.

Concerning the associations between blood proteins and VTE risk, our study is in line with previous studies and revealed additional signals. A proteome-wide MR study based on 81 669 VTE cases of multiple ancestries identified 23 proteins associated with VTE [4], many of which were confirmed in our study, such as those for coagulation FXI, prekallikrein, prothrombin, and protein S with well-defined function in thrombosis. Consistent with findings from another protein-wide study [29], we also identified associations of VTE with genetically predicted levels of kininogen 1 and protein C. For other identified proteins in relation to thrombosis in our MR analysis, we noticed supporting evidences from previous studies on phospholipase C gamma 2 (PLCG2) [30], ANGPT1 [31,32], glycoprotein VI (GPVI) [33], tyrosine kinase Syk (SYK) [34], N-terminal pro-BNP [35], metalloproteinase inhibitor 3 (TIMP-3) [36], extracellular matrix protein 1 (ECM1) [37], ADAMTS-13 [38], protease nexin-1 (SERPINE2) [39], annexin II [40], IL-6 sRa [41], plasma protease C1 inhibitor [42], fibrinogen g-chain dimer [43], Trem-like transcript 1 protein [44], ubiquitin-associated and SH3 domain–containing protein B (UBASH3B, also known as T-cell ubiquitin ligand 2) [45], and regulator of G-protein signaling 18 (RGS18) [46]. Despite limited direct evidence linking VTE with LPR12, this lipid metabolism–related protein has been associated with platelet internalization [47] and vascular endothelial function [48], which thus may be indirectly associated with thrombus formation. Likewise, currently, there is scant evidence concerning the relationship between ADAMTS-like protein 2 and VTE. However, it is worth noting that ADAMTS-like protein 2 belongs to the same protein superfamily as ADAMTS-13 [49], with a clear role in thrombosis. We found few studies on the associations of VTE with LRP4, dermatopontin, endoplasmic reticulum aminopeptidase 2, apolipoprotein L3, microtubule affinity-regulating kinase 3, lactase-phlorizin hydrolase, protein phosphatase 1 regulatory subunit 14A, alpha-(1,6)-fucosyltransferase (FUT8), or anthrax toxin receptor 2. These proteins are related to lipid metabolism, cancer, or immune response. More studies are needed to confirm these associations.

We prioritized protein drug targets by including clinical data from drug databases. Several protein targets, like annexin II, prothrombin, protein C, protein S, ADAMTS-13, coagulation FXI, and fibrinogen g-chain dimer, were reported to have corresponding approved drugs to treat coagulation-related conditions. We also supported GPVI as a highly druggable target with less bleeding risk, which is under investigation in clinical trials [50]. Besides, this study hinted the repurposing values of some proteins approved for other diseases, such as prekallikrein for hereditary angioedema [51] and IL-6 sRa for inflammatory diseases [52], in VTE treatment.

Being different from atherosclerotic cardiovascular outcomes [53], VTE appears to be associated with fewer lifestyle-related factors, mainly obesity [2], smoking [54], and physical inactivity [22], and possibly poor sleep habits [55]. This study found consistent results for obesity [2], smoking [54], and insomnia [55] and reinforced the causality of the associations of physical inactivity and short sleep with VTE for the first time. More interestingly, our 2-stage network MR analysis identified genetically predicted levels of some important proteins mediating these lifestyle–VTE associations. For example, genetically predicted levels of annexin II and coagulation FXI mediated the associations between different modifiable factors (ie, obesity, smoking, and insomnia) with VTE. The 2 proteins shared no clear networks, which explained the associations with lifestyle factors in varying magnitudes. In addition, genetically predicted levels of LPR12, coagulation FXI, and prothrombin consistently mediated most of the association between different obesity indicators and VTE. Some networks between these proteins were observed, which explains a high proportion of the obesity–VTE association explained by these proteins and may shed a light in future studies on interactions of these proteins. Identification of these pathways might not only deepen insight into the pathology underlying the development of VTE, particularly unprovoked VTE from the protein angle, but also guide VTE treatment given that some of these protein mediators are druggable.

The risk of atherosclerotic cardiovascular diseases appears to increase among patients with VTE [10]. Even without direct evidence illuminating detailed pathways linking VTE and excessive risk of cardiovascular comorbidities, this study revealed some proteins, such as genetically predicted levels of LRP12, prothrombin, ANGPT1, LRP4, and IL-6 sRa, shared by etiologies of VTE and other cardiovascular diseases, which may provide clues for cardiovascular risk management among patients with VTE. For example, antiplatelet and anti-inflammation therapy at a low dose may be useful for both cardiovascular disease and VTE prevention. However, these benefits of this strategy should be carefully assessed against the bleeding risk.

The strengths of this study included MR design that minimized confounding and reverse causation, use of the largest GWAS data on VTE to ensure optimal statistical power, consideration of a large number of blood proteins, and investigation using complementary methods. Limitations of the study deserve to be discussed when interpreting the results. First, even though this study involved that many proteins, this analysis might miss proteins without suitable genetic instruments or overlook weak associations due to inadequate power. Second, this study was confined to individuals of European ancestry, which might limit the generalizability of our results to other populations. Third, even though the associations for modifiable factors as the exposures proxied by multiple genetic variants were consistent and with limited indication of unbalanced horizontal pleiotropy in the sensitivity analyses, we could not completely rule out the possibility of horizontal pleiotropy. Fourth, the analyses were based on summary-level data, which did not allow the examination of sex- or age-specific effects or stratification by provoking factors.

In summary, this study suggested that genetically predicted levels of several proteins mediated the positive associations of obesity, smoking, short sleep, and insomnia with VTE risk and some of these protein mediators had highly druggable potentials. These findings may benefit the development of VTE prophylaxis and treatment in high-risk populations.

## Supplementary Material

Supplementary material

## Data Availability

The study was based on publicly available summary-level data that can be downloaded from the cited genome-wide association studies.

## Code Availability

The study was based on publicly available summary-level data that can be downloaded from the cited genome-wide association studies.

Publicly available software was used to perform the analyses. Code is available from the corresponding author upon reasonable request.

## References

[1] Lutsey PL, Zakai NA (2023). Epidemiology and prevention of venous thromboembolism. Nat Rev Cardiol.

[2] Yuan S, Bruzelius M, Xiong Y, Håkansson N, Åkesson A, Larsson SC (2021). Overall and abdominal obesity in relation to venous thromboembolism. J Thromb Haemost.

[3] Folsom AR, Cushman M (2020). Exploring opportunities for primary prevention of unprovoked venous thromboembolism: ready for prime time?. J Am Heart Assoc.

[4] Thibord F, Klarin D, Brody JA, Chen MH, Levin MG, Chasman DI, Goode EL, Hveem K, Teder-Laving M, Martinez-Perez A, Aïssi D (2022). Cross-ancestry investigation of venous thromboembolism genomic predictors. Circulation.

[5] Yuan S, Titova OE, Zhang K, Gou W, Schillemans T, Natarajan P, Chen J, Li X, Åkesson A, Bruzelius M, Klarin D (2023). Plasma protein and venous thromboembolism: prospective cohort and Mendelian randomisation analyses. Br J Haematol.

[6] Watanabe K, Wilmanski T, Diener C, Earls JC, Zimmer A, Lincoln B, Hadlock JJ, Lovejoy JC, Gibbons SM, Magis AT, Hood L (2023). Multiomic signatures of body mass index identify heterogeneous health phenotypes and responses to a lifestyle intervention. Nat Med.

[7] Khan F, Tritschler T, Kimpton M, Wells PS, Kearon C, Weitz JI, Büller HR, Raskob GE, Ageno W, Couturaud F, Prandoni P (2021). Long-term risk for major bleeding during extended oral anticoagulant therapy for first unprovoked venous thromboembolism: a systematic review and meta-analysis. Ann Intern Med.

[8] Donzé J, Rodondi N, Waeber G, Monney P, Cornuz J, Aujesky D (2012). Scores to predict major bleeding risk during oral anticoagulation therapy: a prospective validation study. Am J Med.

[9] Chen J, Xu F, Ruan X, Sun J, Zhang Y, Zhang H, Zhao J, Zheng J, Larsson SC, Wang X, Li X (2023). Therapeutic targets for inflammatory bowel disease: proteome-wide Mendelian randomization and colocalization analyses. EBioMedicine.

[10] Noumegni SR, Hoffmann C, Tromeur C, Lacut K, Didier R, Couturaud F, Bressollette L (2021). Frequency and incidence of arterial events in patients with venous thromboembolism compared to the general population: a systematic review and meta-analysis of cohort studies. Thromb Res.

[11] Burgess S, Thompson SG (2015). Mendelian randomization: methods for using genetic variants in causal estimation.

[12] Holmes MV, Ala-Korpela M, Smith GD (2017). Mendelian randomization in cardiometabolic disease: challenges in evaluating causality. Nat Rev Cardiol.

[13] Zheng J, Haberland V, Baird D, Walker V, Haycock PC, Hurle MR, Gutteridge A, Erola P, Liu Y, Luo S, Robinson J (2020). Phenome-wide Mendelian randomization mapping the influence of the plasma proteome on complex diseases. Nat Genet.

[14] Hemani G, Bowden J, Davey Smith G (2018). Evaluating the potential role of pleiotropy in Mendelian randomization studies. Hum Mol Genet.

[15] Ferkingstad E, Sulem P, Atlason BA, Sveinbjornsson G, Magnusson MI, Styrmisdottir EL, Gunnarsdottir K, Helgason A, Oddsson A, Halldorsson BV, Jensson BO (2021). Large-scale integration of the plasma proteome with genetics and disease. Nat Genet.

[16] Pietzner M, Wheeler E, Carrasco-Zanini J, Cortes A, Koprulu M, Wörheide MA, Oerton E, Cook J, Stewart ID, Kerrison ND, Luan J (2021). Mapping the proteo-genomic convergence of human diseases. Science.

[17] Sun BB, Chiou J, Traylor M, Benner C, Hsu YH, Richardson TG, Surendran P, Mahajan A, Robins C, Vasquez-Grinnell SG, Hou L (2023). Plasma proteomic associations with genetics and health in the UK Biobank. Nature.

[18] Ghouse J, Tragante V, Ahlberg G, Rand SA, Jespersen JB, Leinø JB, Vissing EB, Trudsø CR, Jonsdottir I, Banasik K, Brunak S (2023). Genome-wide meta-analysis identifies 93 risk loci and enables risk prediction equivalent to monogenic forms of venous thromboembolism. Nat Genet.

[19] Cheng YJ, Liu ZH, Yao FJ, Zeng WT, Zheng DD, Dong YG, Wu SH (2013). Current and former smoking and risk for venous thromboembolism: a systematic review and meta-analysis. PLoS Med.

[20] Johansson M, Johansson L, Wennberg M, Lind M (2019). Alcohol consumption and risk of first-time venous thromboembolism in men and women. Thromb Haemost.

[21] Enga KF, Braekkan SK, Hansen-Krone IJ, Wilsgaard T, Hansen JB (2011). Coffee consumption and the risk of venous thromboembolism: the Tromsø study. J Thromb Haemost.

[22] Yuan S, Bruzelius M, Hékansson N, Åkesson A, Larsson SC (2021). Lifestyle factors and venous thromboembolism in two cohort studies. Thromb Res.

[23] Kubota Y, Cushman M, Zakai N, Rosamond WD, Folsom AR (2018). TV viewing and incident venous thromboembolism: the Atherosclerotic Risk in Communities Study. J Thromb Thrombolysis.

[24] St-Onge MP, Grandner MA, Brown D, Conroy MB, Jean-Louis G, Coons M, Bhatt DL, American Heart Association Obesity, Behavior Change, Diabetes, and Nutrition Committees of the Council on Lifestyle and Cardiometabolic Health; Council on Cardiovascular Disease in the Young; Council on Clinical Cardiology; and Stroke Council (2016). Sleep duration and quality: impact on lifestyle behaviors and cardiometabolic health: a scientific statement from the American Heart Association. Circulation.

[25] Chung WS, Chen YF, Lin CL, Chang SN, Hsu WH, Kao CH (2015). Sleep disorders increase the risk of venous thromboembolism in individuals without sleep apnea: a nationwide population-based cohort study in Taiwan. Sleep Med.

[26] Ghoussaini M, Mountjoy E, Carmona M, Peat G, Schmidt EM, Hercules A, Fumis L, Miranda A, Carvalho-Silva D, Buniello A, Burdett T (2021). Open targets genetics: systematic identification of trait-associated genes using large-scale genetics and functional genomics. Nucleic Acids Res.

[27] Larsson SC, Woolf B, Gill D (2023). Appraisal of the causal effect of plasma caffeine on adiposity, type 2 diabetes, and cardiovascular disease: two sample Mendelian randomisation study. BMJ Med.

[28] Warde-Farley D, Donaldson SL, Comes O, Zuberi K, Badrawi R, Chao P, Franz M, Grouios C, Kazi F, Lopes CT, Maitland A (2010). The GeneMANIA prediction server: biological network integration for gene prioritization and predicting gene function. Nucleic Acids Res.

[29] Li H, Zhang Z, Qiu Y, Weng H, Yuan S, Zhang Y, Zhang Y, Xi L, Xu F, Ji X, Hao R (2023). Proteome-wide Mendelian randomization identifies causal plasma proteins in venous thromboembolism development. J Hum Genet.

[30] Elvers M, Pozgaj R, Pleines I, May F, Kuijpers MJ, Heemskerk JM, Yu P, Nieswandt B (2010). Platelet hyperreactivity and a prothrombotic phenotype in mice with a gain-of-function mutation in phospholipase Cgamma2. J Thromb Haemost.

[31] Gangaraju R, Song J, Kim SJ, Tashi T, Reeves BN, Sundar KM, Thiagarajan P, Prchal JT (2020). Thrombotic, inflammatory, and HIF-regulated genes and thrombosis risk in polycythemia vera and essential thrombocythemia. Blood Adv.

[32] Kreft IC, Huisman EJ, Cnossen MH, van Alphen FPJ, van der Zwaan C, van Leeuwen K, van Spaendonk R, Porcelijn L, Veen CSB, van den Biggelaar M, de Haas M (2023). Proteomic landscapes of inherited platelet disorders with different etiologies. J Thromb Haemost.

[33] Perrella G, Nagy M, Watson SP, Heemskerk JWM (2021). Platelet GPVI (glycoprotein VI) and thrombotic complications in the venous system. Arterioscler Thromb Vasc Biol.

[34] Reilly MP, Sinha U, André P, Taylor SM, Pak Y, Deguzman FR, Nanda N, Pandey A, Stolla M, Bergmeier W, McKenzie SE (2011). PRT-060318, a novel Syk inhibitor, prevents heparin-induced thrombocytopenia and thrombosis in a transgenic mouse model. Blood.

[35] Alonso-Martínez JL, Urbieta-Echezarreta M, Anniccherico-Sánchez FJ, Abínzano-Guillén ML, Garcia-Sánchotena JL (2009). N-terminal pro-B-type natriuretic peptide predicts the burden of pulmonary embolism. Am J Med Sci.

[36] Santos-Martínez MJ, Medina C, Jurasz P, Radomski MW (2008). Role of metalloproteinases in platelet function. Thromb Res.

[37] Bergmeier W, Hynes RO (2012). Extracellular matrix proteins in hemostasis and thrombosis. Cold Spring Harb Perspect Biol.

[38] Muia J, Zhu J, Gupta G, Haberichter SL, Friedman KD, Feys HB, Deforche L, Vanhoorelbeke K, Westfield LA, Roth R, Tolia NH (2014). Allosteric activation of ADAMTS13 by von Willebrand factor. Proc Natl Acad Sci U S A.

[39] Bouton MC, Boulaftali Y, Richard B, Arocas V, Michel JB, Jandrot-Perrus M (2012). Emerging role of serpinE2/protease nexin-1 in hemostasis and vascular biology. Blood.

[40] Cañas F, Simonin L, Couturaud F, Renaudineau Y (2015). Annexin A2 autoantibodies in thrombosis and autoimmune diseases. Thromb Res.

[41] Senchenkova EY, Komoto S, Russell J, Almeida-Paula LD, Yan LS, Zhang S, Granger DN (2013). Interleukin-6 mediates the platelet abnormalities and thrombogenesis associated with experimental colitis. Am J Pathol.

[42] Grover SP, Kawano T, Wan J, Tanratana P, Polai Z, Shim YJ, Snir O, Brækkan S, Dhrolia S, Kasthuri RR, Bendapudi PK (2023). C1 inhibitor deficiency enhances contact pathway-mediated activation of coagulation and venous thrombosis. Blood.

[43] Grünbacher G, Weger W, Marx-Neuhold E, Pilger E, Köppel H, Wascher T, März W, Renner W (2007). Thefibrinogen gamma (FGG) 10034C>T polymorphism is associated with venous thrombosis. Thromb Res.

[44] Smith CW, Raslan Z, Parfitt L, Khan AO, Patel P, Senis YA, Mazharian A (2018). TREM-like transcript 1: a more sensitive marker of platelet activation than P-selectin in humans and mice. Blood Adv.

[45] Zhou Y, Abraham S, Andre P, Edelstein LC, Shaw CA, Dangelmaier CA, Tsygankov AY, Kunapuli SP, Bray PF, McKenzie SE (2015). Anti-miR-148a regulates platelet FcγRIIA signaling and decreases thrombosis in vivo in mice. Blood.

[46] Alshbool FZ, Karim ZA, Vemana HP, Conlon C, Lin OA, Khasawneh FT (2015). The regulator of G-protein signaling 18 regulates platelet aggregation, hemostasis and thrombosis. Biochem Biophys Res Commun.

[47] Miao S, Zhang Q, Ding W, Hou B, Su Z, Li M, Yang L, Zhang J, Chang W, Wang J (2023). Platelet internalization mediates ferroptosis in myocardial infarction. Arterioscler Thromb Vasc Biol.

[48] Debette S, Visvikis-Siest S, Chen MH, Ndiaye NC, Song C, Destefano A, Safa R, Azimi Nezhad M, Sawyer D, Marteau JB, Xanthakis V (2011). Identification of cis- and trans-acting genetic variants explaining up to half the variation in circulating vascular endothelial growth factor levels. Circ Res.

[49] Le Goff C, Cormier-Daire V (2011). The ADAMTS(L) family and human genetic disorders. Hum Mol Genet.

[50] Mackman N, Bergmeier W, Stouffer GA, Weitz JI (2020). Therapeutic strategies for thrombosis: new targets and approaches. Nat Rev Drug Discov.

[51] Fijen LM, Riedl MA, Bordone L, Bernstein JA, Raasch J, Tachdjian R, Craig T, Lumry WR, Manning ME, Alexander VJ, Newman KB (2022). Inhibition of prekallikrein for hereditary angioedema. N Engl J Med.

[52] Kang S, Tanaka T, Narazaki M, Kishimoto T (2019). Targeting interleukin-6 signaling in clinic. Immunity.

[53] Yuan S, Mason AM, Burgess S, Larsson SC (2022). Differentiating associations of glycemic traits with atherosclerotic and thrombotic outcomes: Mendelian randomization investigation. Diabetes.

[54] Larsson SC, Mason AM, Bäck M, Klarin D, Damrauer SM, Michaëlsson K, Burgess S, Million Veteran Program (2020). Genetic predisposition to smoking in relation to 14 cardiovascular diseases. Eur Heart J.

[55] Yuan S, Mason AM, Burgess S, Larsson SC (2021). Genetic liability to insomnia in relation to cardiovascular diseases: a Mendelian randomisation study. Eur J Epidemiol.

