# Supplementary material for: Proteomic insights into modifiable risk of venous thromboembolism and cardiovascular comorbidities
Source: J Thromb Haemost. Author manuscript; Available in PMC 2024 Mar 1. (PMC7615672; doi:10.1016/j.jtha.2023.11.013)
Supplement: Supplementary material [file EMS193801-supplement-Supplementary_material.pdf]

Supplementary methods, tables, and figures for

Proteomic insights into modifiable risk of venous thromboembolism and cardiovascular comorbidities

Table of Contents

Supplementary methods **2**

Polygenic risk score-Phenome-wide association study in UK Biobank **2**

Colocalization analysis **2**

Supplementary Tables 1-13 .... **3**

Supplementary Figure 1. The networks of VTE-associated proteins. **4**

Supplementary Figure 2. Results of VTE PRS-PheWAS in the UK Biobank. After Bonferroni correction, this analysis identified 38 clinical outcomes associated with polygenic risk score of VTE. By clustering the outcomes in groups, the identified outcomes majorly belong to the disease of circulatory system and hematopoietic disease. **5**

Supplementary Figure 3. Cardiovascular effects of proteins shared by VTE and studied cardiovascular comorbidities in Open Targets Genetics database. **8**

References **9**

Supplementary methods

Polygenic risk score-Phenome-wide association study in UK Biobank

We selected SNPs associated with VTE at the $P < 5\times10^{-8}$ in the GWAS meta-analysis[1] and without linkage disequilibrium (r^2 <0.01) to construct the polygenic risk score (PRS). The weighted PRS was created by summing the number of VTE-liability-increasing alleles for each SNP weighted by effect size on VTE liability and then adding this weighted score for all used SNPs. We performed a PRS-PheWAS in the UK Biobank to explore the comorbidities associated with VTE. The PRS-PheWAS was based on 1186 phenotypes with cases > 120. The phenotypes were defined by the PheCODE schema based on ICD-9 and -10 codes.[2] The associations were estimated by a logistic regression with adjustment for age[2], sex, assessment center, and the first 10 principal components. Bonferroni method was used to correct for multiple testing ($P < 4.2\times10^{-5}$). Details of the PRS-PheWAS can be found elsewhere.[3]

Colocalization analysis

We conducted colocalization analysis using the coloc R package[4] to test the associations between proteins and VTE were driven by linkage disequilibrium. For each locus, the Bayesian method assessed the support for the following five exclusive hypotheses: 1) no association with either trait; 2) association with trait 1 only; 3) association with trait 2 only; 4) both traits are associated, but distinct causal variants were for two traits; and 5) both traits are associated, and the same shares causal variant for both traits. The analysis provides posterior probabilities for each hypothesis testing (H0, H1, H2, H3, and H4). We set prior probabilities of the SNP being associated with trait 1 only (p1) at 1×10^{-4}; the probability of the SNP being associated with trait 2 only (p2) at 1×10^{-4}; and the probability of the SNP being associated with both traits (p12) at 1×10^{-5} two signals were considered to have strong evidence of colocalization if the posterior probability for shared causal variants (PH4) was ≥0.8.

The colocalization analysis was based on summary-level statistics of genetic associations with levels of 4907 circulating proteins from a large-scale protein quantitative trait loci (pQTL) study in 35,559 Icelanders.[5] Proteomic profiling was performed by a multiplexed, modified aptamer-based binding assay (SOMAscan version 4). The levels of protein were rank-inverse normal transformed by age and sex. The residuals were standardized using rank-inverse normal transformation and the standardized values were treated as phenotypes in the genome-wide association analyses under the BOLT-LMM linear mixed model.

Supplementary Tables 1-13

Supplementary tables 1-8 have been uploaded in the OSF data respiratory and can be obtained via the link (https://osf.io/hkqy5/).

ST1. Data sources for included studies
ST2. Associations of 1151 proteins with VTE risk
ST3. Proteins with strong colocalization support with VTE
ST4. Replicated associations of 31 proteins with VTE using IVs from the Fenland study
ST5. Replicated associations of 9 proteins with VTE using IVs from UKB-PPP
ST6. Associations of genetic liability to VTE with the levels of identified blood proteins in the reverse MR analysis
ST7. Druggability assessment of identified VTE-assocaited proteins
ST8. Associations of 15 modifiable factors with venous thromboembolism
ST9. Associations of genetic liability to VTE with modifiable risk factors in the reverse MR analysis
ST10. Associations of VTE-associated modifiable factors with VTE-associated blood proteins
ST11. Associations of VTE-associated modifiable factors with VTE-associated blood proteins in Fenland
ST12. Estimated mediation of blood proteins in the associations between modifiable risk factors and VTE risk
ST13. Associations of genetically proxied VTE with cardiovascular diseases

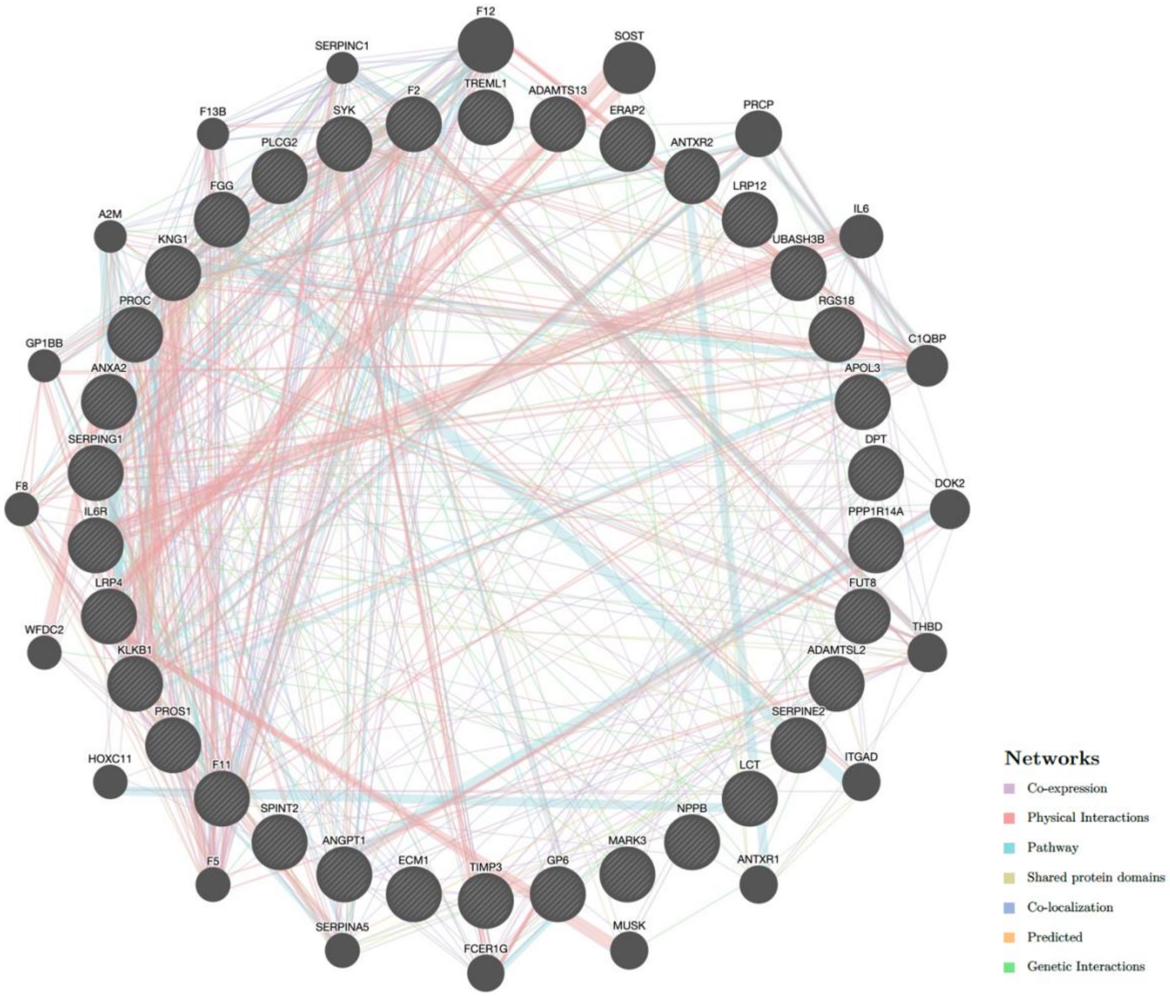

Supplementary Figure 1. The networks of VTE-associated proteins.

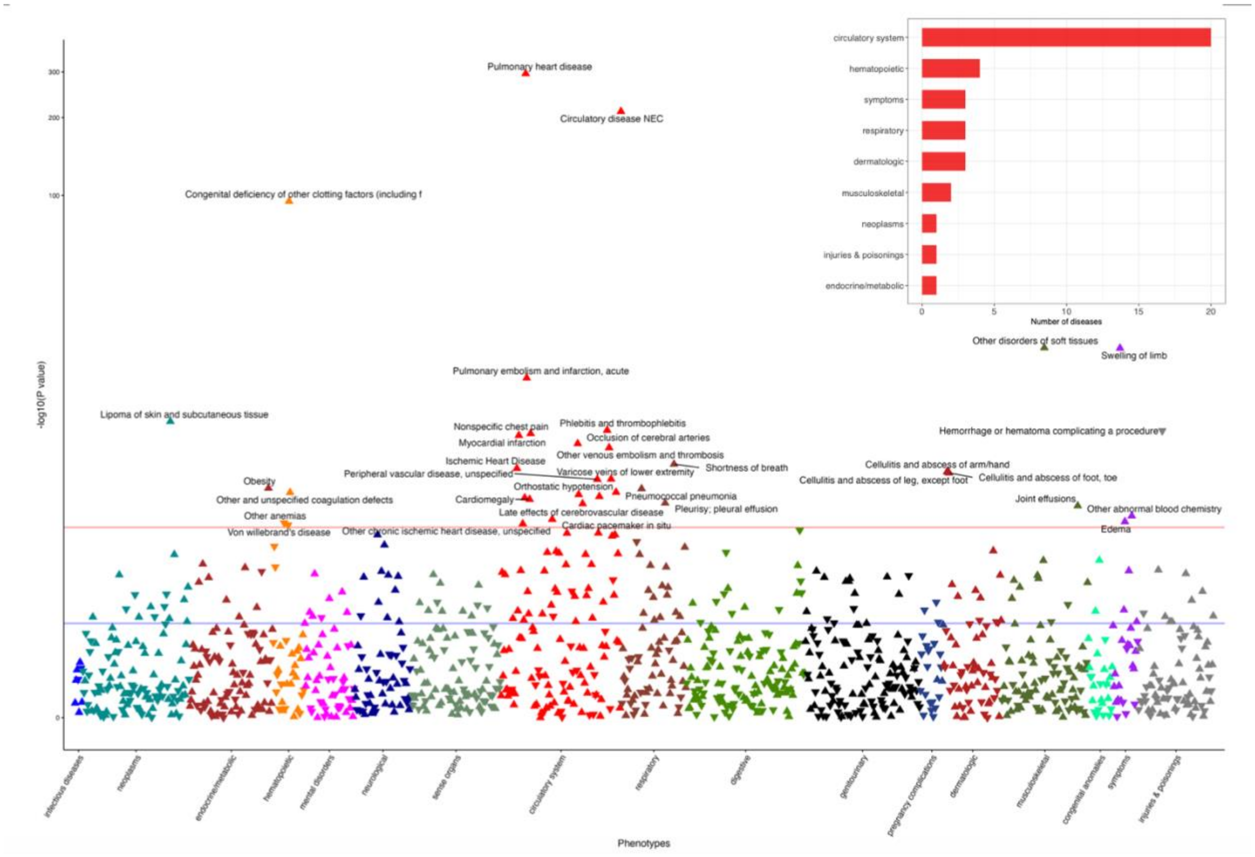

Supplementary Figure 2. Results of VTE PRS-PheWAS in the UK Biobank. After Bonferroni correction, this analysis identified 38 clinical outcomes associated with polygenic risk score of VTE. By clustering the outcomes in groups, the identified outcomes majorly belong to the disease of circulatory system and hematopoietic disease.

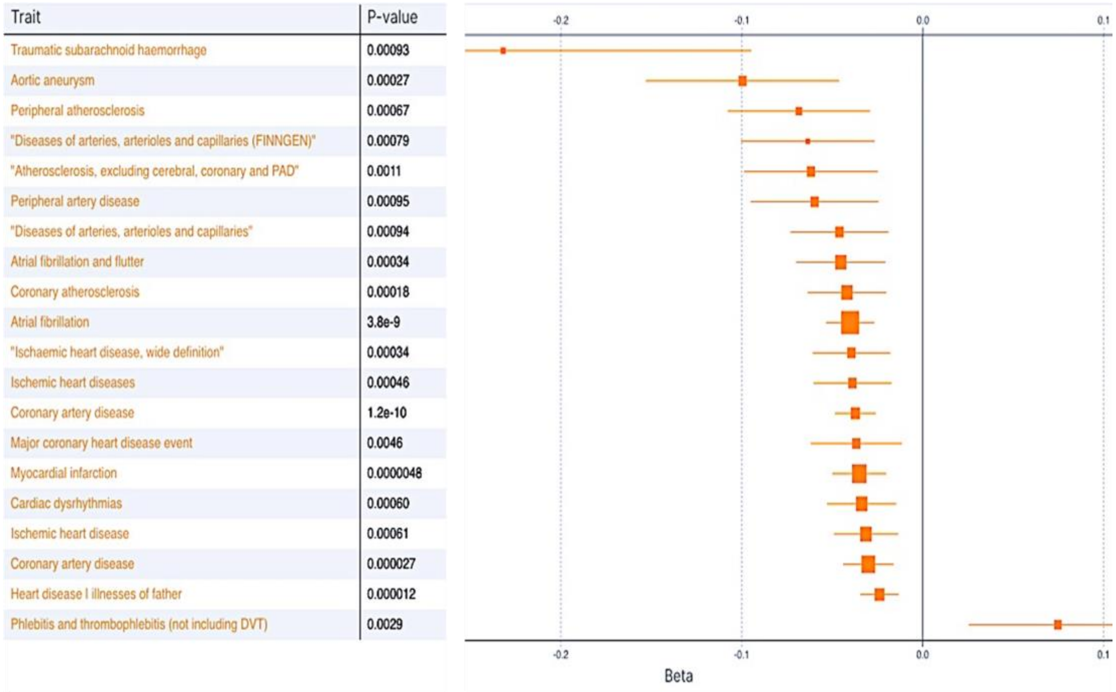

A. Cardiovascular effects of IL-6 sRa (rs12126142; effect allele = A) in Open Targets Genetics.

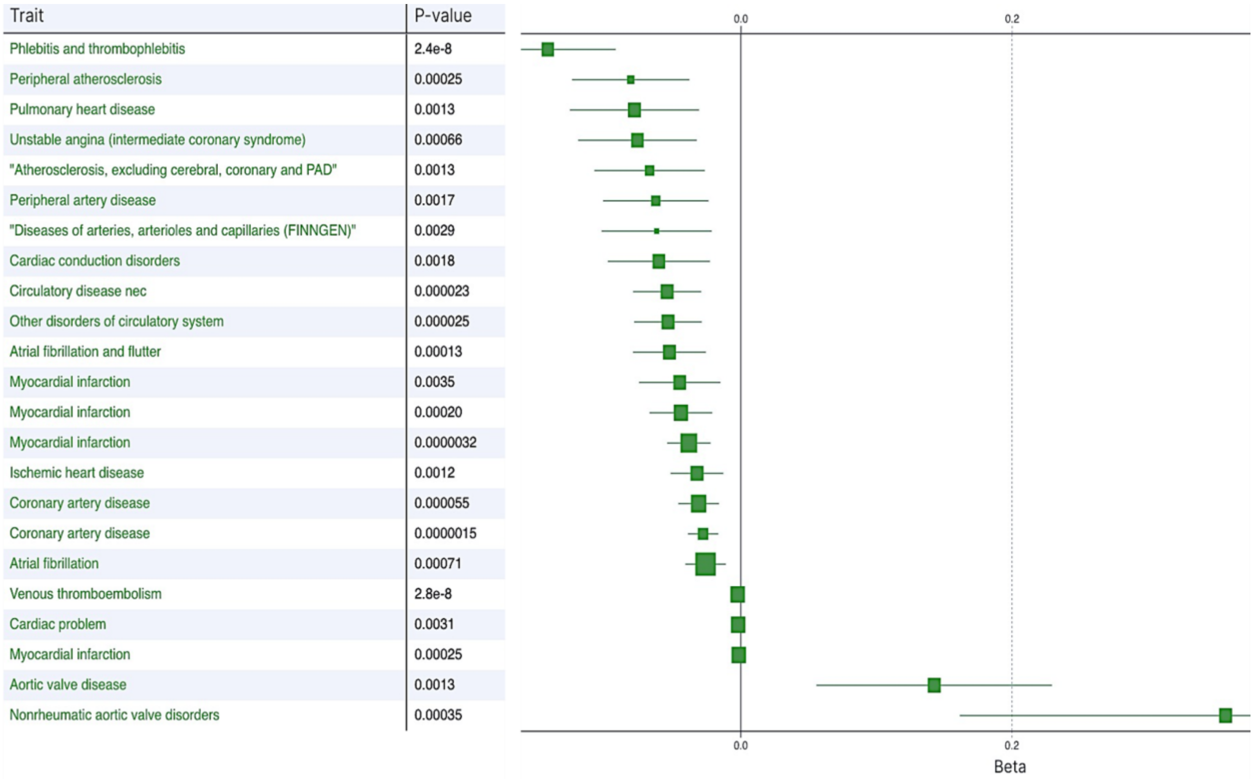

B. Cardiovascular effects of LRP12 (rs6993770; effect allele = T) in Open Targets Genetics.

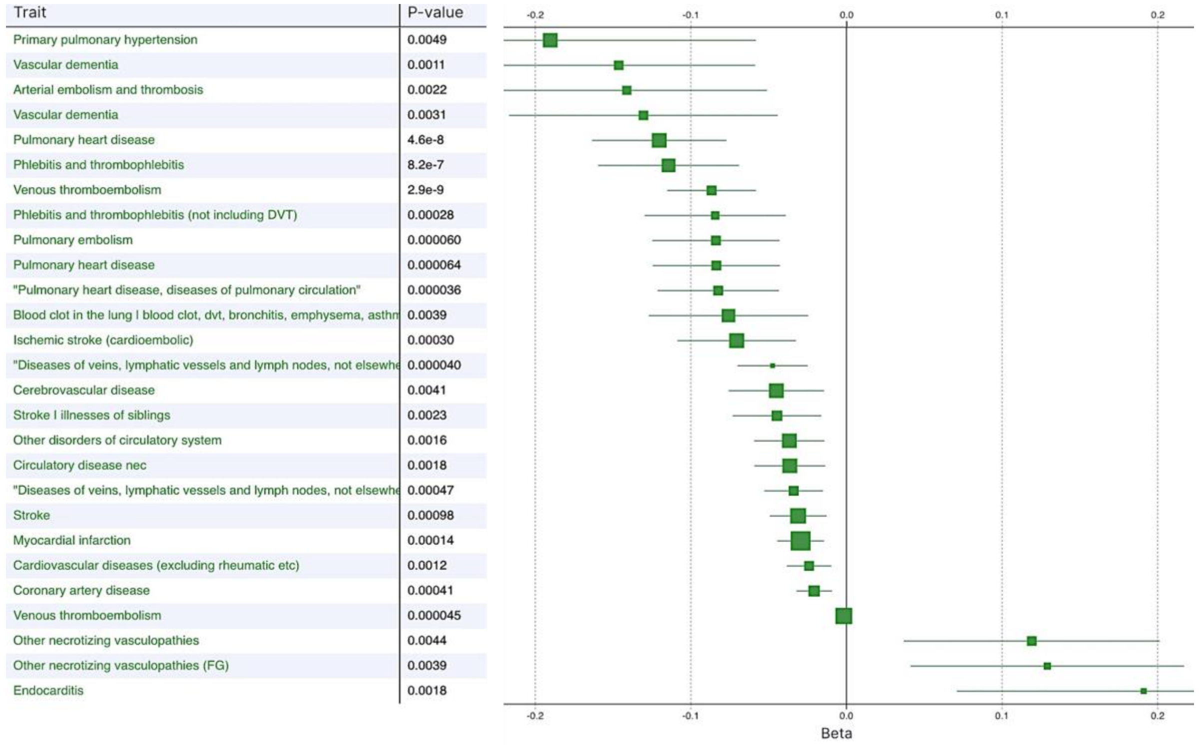

C. Cardiovascular effects of prothrombin (rs3136516; effect allele = A) in Open Targets Genetics.

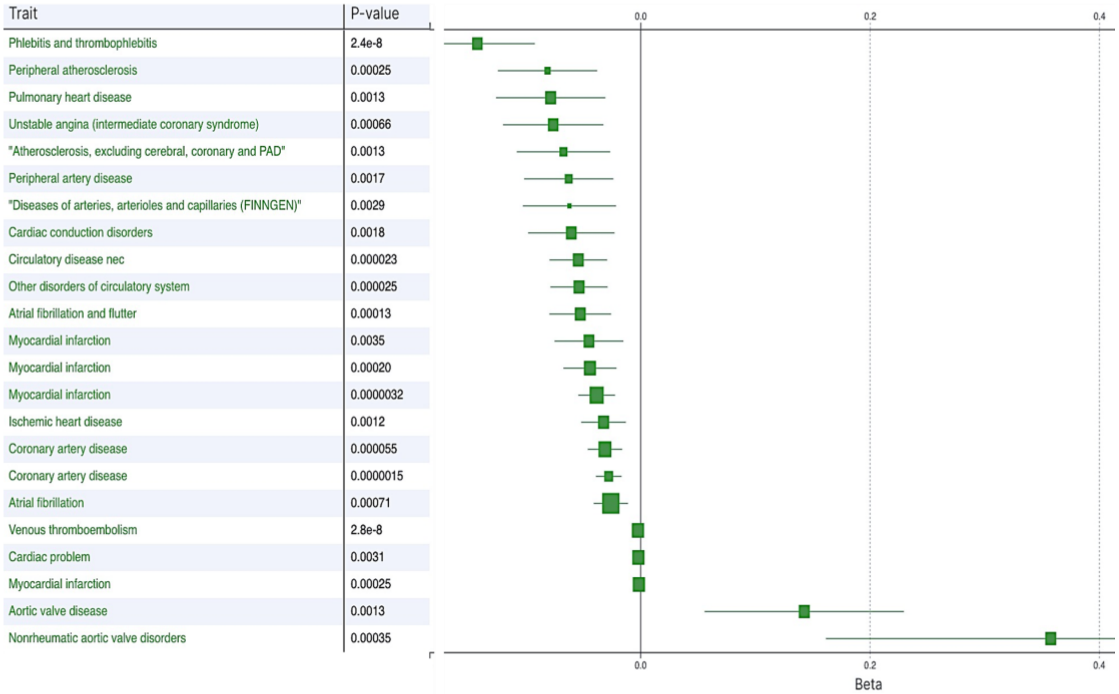

D. Cardiovascular effects of angiopoietin-1 (rs6993770; effect allele = T) in Open Targets Genetics.

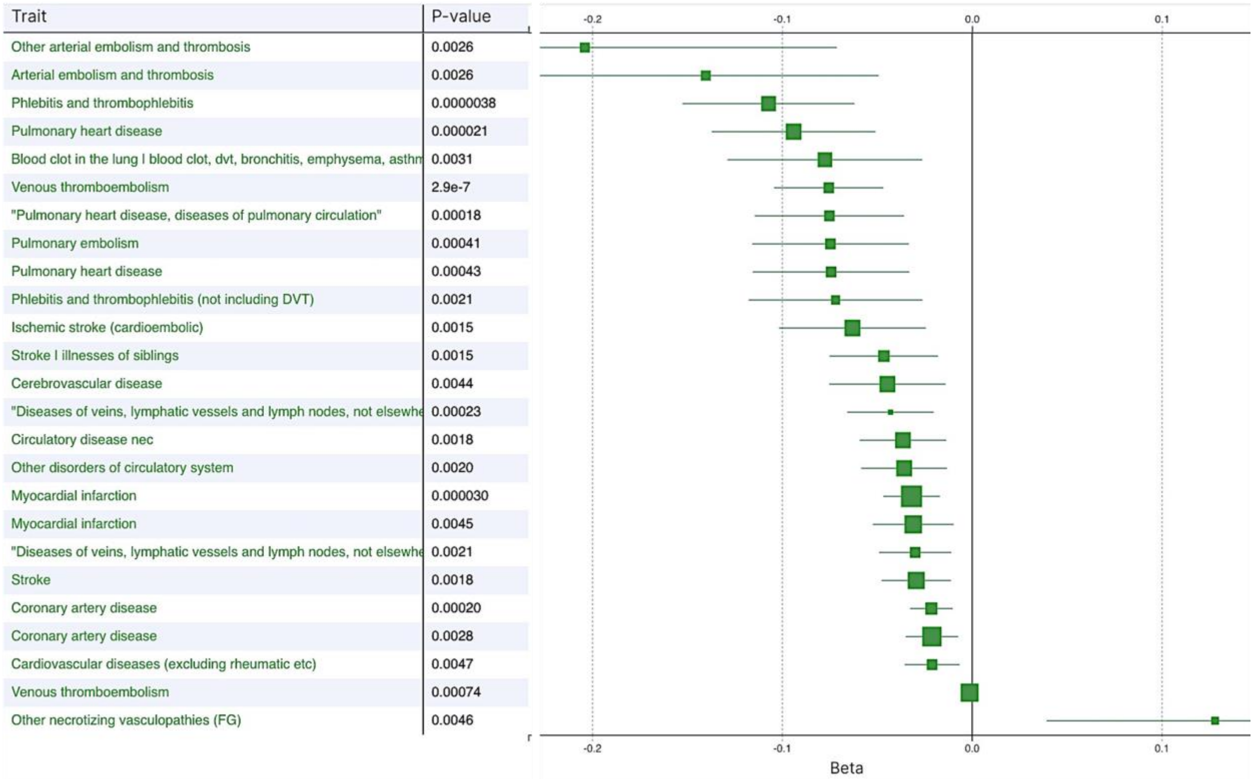

E. Cardiovascular effects of LRP4 (rs2306029; effect allele = C) in Open Targets Genetics.

Supplementary Figure 3. Cardiovascular effects of proteins shared by VTE and studied cardiovascular comorbidities in Open Targets Genetics database.

References

1. Ghouse J, Tragante V, Ahlberg G, Rand SA, Jespersen JB, Leinøe EB, Vissing CR, Trudsø L, Jonsdottir I, Banasik K, Brunak S, Ostrowski SR, Pedersen OB, Sørensen E, Erikstrup C, Bruun MT, Nielsen KR, Køber L, Christensen AH, Iversen K, Jones D, Knowlton KU, Nadauld L, Halldorsson GH, Ferkingstad E, Olafsson I, Gretarsdottir S, Onundarson PT, Sulem P, Thorsteinsdottir U, Thorgeirsson G, Gudbjartsson DF, Stefansson K, Holm H, Olesen MS, Bundgaard H. Genome-wide meta-analysis identifies 93 risk loci and enables risk prediction equivalent to monogenic forms of venous thromboembolism. Nat Genet 2023;**55**(3):399-409.

2. Denny JC, Bastarache L, Ritchie MD, Carroll RJ, Zink R, Mosley JD, Field JR, Pulley JM, Ramirez AH, Bowton E, Basford MA, Carrell DS, Peissig PL, Kho AN, Pacheco JA, Rasmussen LV, Crosslin DR, Crane PK, Pathak J, Bielinski SJ, Pendergrass SA, Xu H, Hindorff LA, Li R, Manolio TA, Chute CG, Chisholm RL, Larson EB, Jarvik GP, Brilliant MH, McCarty CA, Kullo IJ, Haines JL, Crawford DC, Masys DR, Roden DM. Systematic comparison of phenome-wide association study of electronic medical record data and genome-wide association study data. Nat Biotechnol 2013;**31**(12):1102-10.

3. Yuan S, Wang L, Sun J, Yu L, Zhou X, Yang J, Zhu Y, Gill D, Burgess S, Denny JC, Larsson SC, Theodoratou E, Li X. Genetically predicted sex hormone levels and health outcomes: phenome-wide Mendelian randomization investigation. Int J Epidemiol 2022.

4. Giambartolomei C, Vukcevic D, Schadt EE, Franke L, Hingorani AD, Wallace C, Plagnol V. Bayesian test for colocalisation between pairs of genetic association studies using summary statistics. PLoS Genet 2014;**10**(5):e1004383.

5. Ferkingstad E, Sulem P, Atlason BA, Sveinbjornsson G, Magnusson MI, Styrmisdottir EL, Gunnarsdottir K, Helgason A, Oddsson A, Halldorsson BV, Jensson BO, Zink F, Halldorsson GH, Masson G, Arnadottir GA, Katrinardottir H, Juliusson K, Magnusson MK, Magnusson OT, Fridriksdottir R, Saevarsdottir S, Gudjonsson SA, Stacey SN, Rognvaldsson S, Eiriksdottir T, Olafsdottir TA, Steinthorsdottir V, Tragante V, Ulfarsson MO, Stefansson H, Jonsdottir I, Holm H, Rafnar T, Melsted P, Saemundsdottir J, Norddahl GL, Lund SH, Gudbjartsson DF, Thorsteinsdottir U, Stefansson K. Large-scale integration of the plasma proteome with genetics and disease. Nat Genet 2021;**53**(12):1712-1721.
